# Supplementary material for: Using Human-Centered Design in Community-Based Public Health Research: Insights from the ECHO Study on COVID-19 Vaccine Hesitancy in Montreal, Canada
Source: Int J Environ Res Public Health. 2025 Jan 30;22(2):198. doi: 10.3390/ijerph22020198 (PMC11855836; doi:10.3390/ijerph22020198)
Supplement: Supplementary file 1 [file ijerph-22-00198-s001.zip › ijerph-3300713-supplementary.pdf]

Projet ECHO Final Report

November 2022

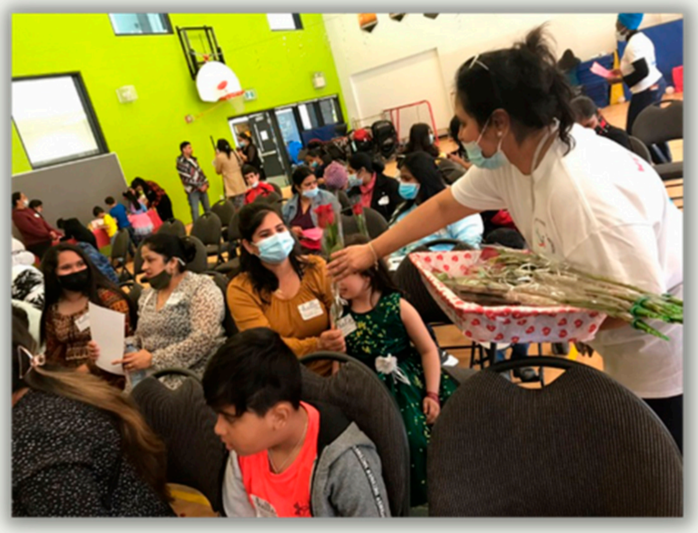

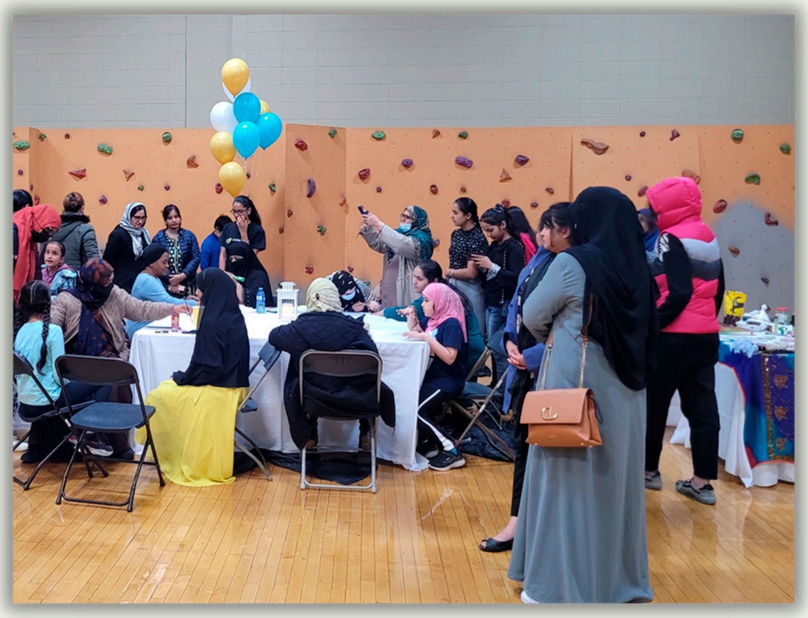

ECHO Project

ECHO is a community-based participatory research project led by 16 parent and youth community researchers from Montreal North and Parc Extension, supported by health researchers and design thinking specialists.

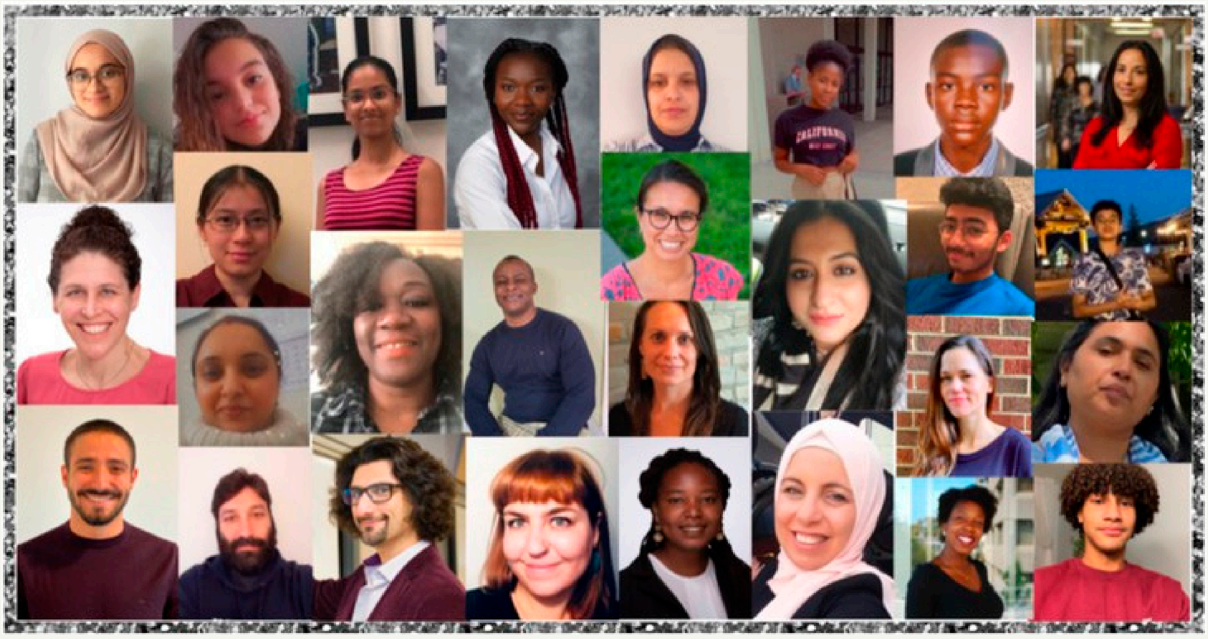

Between December 2021 and September 2022, we:

- Explored how parents and adolescents make decisions about COVID-19 vaccination in the communities of Montreal-Nord and Parc Extension
- Designed and implemented strategies to enhance vaccine confidence and support youth and families experiencing negative impacts of the pandemic

We thank you for your support and are excited to share our findings!

Table of Contents

01 **Introduction**

1.1 Parc-Extension and Montreal-Nord: two vibrant diverse Montreal communities

1.2 Projet ECHO: purpose and design

02 **Youths' Projects**

2.1 Perspectives on COVID-19 vaccination

2.2 Les Ados du Nord: My Choice for My Community

2.3 Team APEX (ados of Parc Ex): Using educational gaming to combat misinformation

03 **Parents' Projects**

3.1 Perspectives on COVID-19 vaccination for their children

3.2 Vision of Parc Ex Mothers: Fostering community trust

3.3 ECHO du Nord parents: Bringing parents' voices into child health initiatives in Montreal-Nord

04 **Reflections & Recommendations**

4.1 Lessons from using Human Centred Design in community participatory health research

4.2 Our challenges & recommendations

Chapter 1

01

Introduction

1.1 Parc-Extension and Montreal-Nord: two vibrant diverse Montreal communities
1.2 Projet ECHO: purpose and design

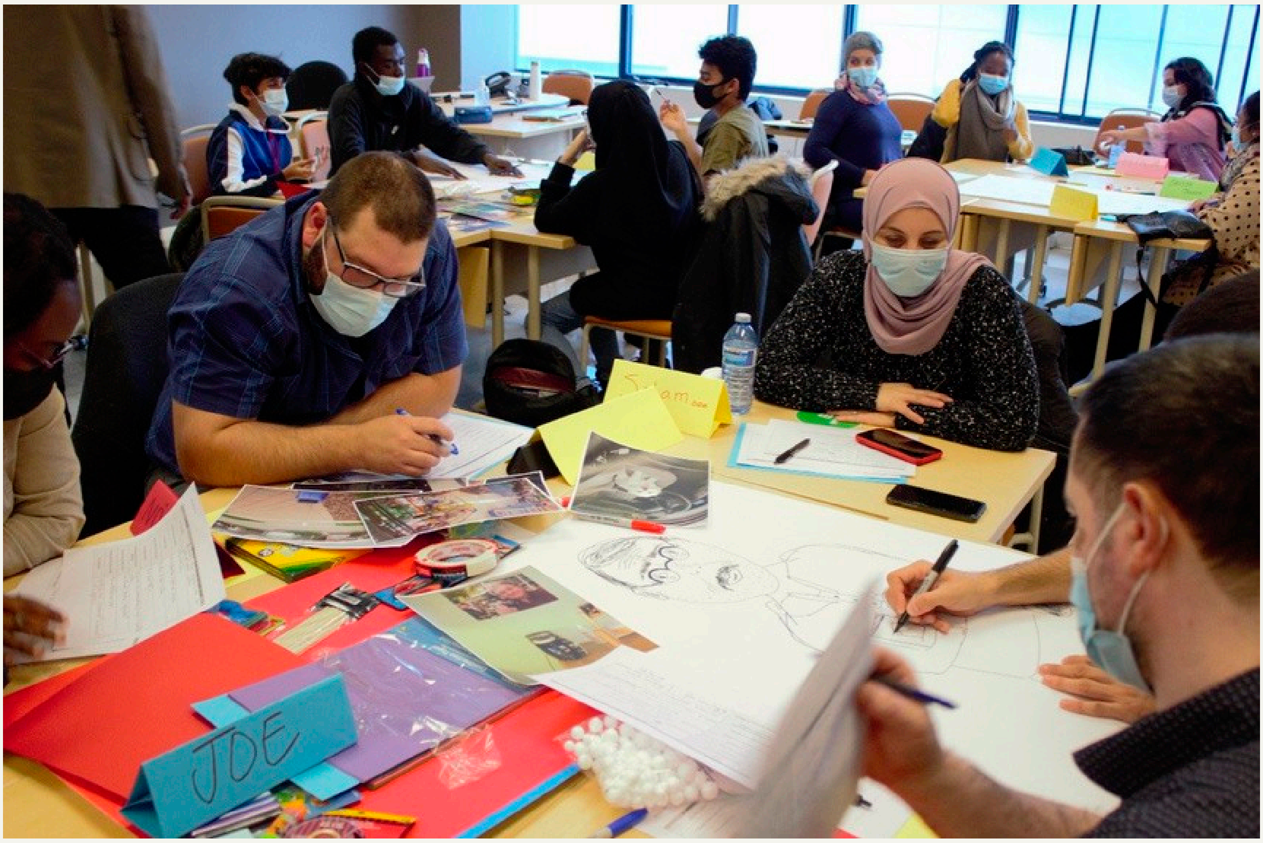

1.1 Two vibrant and diverse Montreal communities

Parc Extension

- Home to the largest immigrant population in Montreal, Parc Ex showcases its ethnocultural diversity through community, food, clothing, art, and places of workshop.
- One of the poorest and most densely populated neighbourhoods in Canada: income insecurity, inaccess to healthcare services and the lack of affordable quality housing and green spaces are major concerns.

> *I moved to Park Ex in 2013. It was the first time in my life as a Montrealer that a neighbour not only came over to introduce themselves, but also to introduce me to the other neighbours. And it has to be said that the first person to ever go out of their way to welcome me in Montréal was an immigrant."*
>
> -Source: Glimpses of Parc-Ex

- The COVID-19 pandemic exacerbated existing social inequities in Parc Ex
- A lack of socioculturally and linguistically appropriate public health messaging about the COVID-19 pandemic likely contributed to slower COVID-19 vaccination uptake in Parc Ex compared to other Montreal neighbourhoods

Les Montréalais démunis deux fois plus touchés

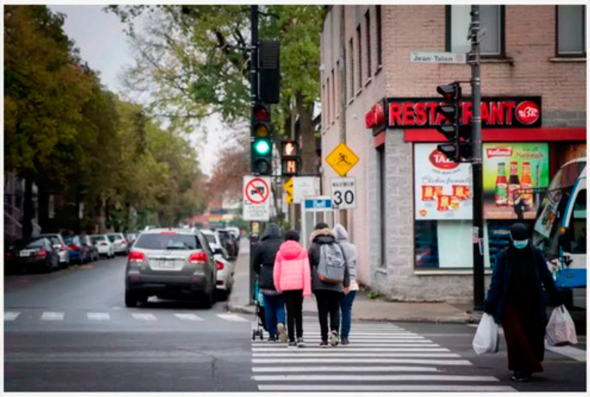

PHOTO SARAH MONGEAU-BIRKETT, LA PRESSE
Le quartier Parc-Extension, à Montréal

Les Montréalais qui résident dans les secteurs très défavorisés de la ville ont été deux fois plus touchés par la COVID-19 que ceux qui vivent dans les quartiers très favorisés. La mortalité y est également deux fois plus élevée.

Montreal-Nord

- A large and racially diverse neighbourhood, home to one of Canada's largest Haitian communities and sizeable Arabic and Latin American populations
- Among Montreal's poorest neighbourhoods, with many residents working in low-paying essential service jobs
- Per capita rate of COVID-19 almost double the city of Montreal's rate, making it one of Canada's most affected communities
- COVID-19 vaccination rates, including among children and adolescents, are among the lowest in Montreal

https://www.centraide-mtl.org/wp-content/uploads/2021/01/Portrait-Montreal-Nord-2017-2018.pdf
https://www.lapresse.ca/covid-19/2021-10-22/les-montrealais-demunis-deux-fois-plus-touches.php#

1.2 Projet ECHO: Purpose and Design

Project ECHO (Étude communautaire sur l'hésitation à la vaccination contre la COVID-19) is a **community-based participatory research study** that aimed to:

- Understand local perspectives around COVID-19 vaccination,
- Co-develop and implement strategies with community researchers to enhance vaccine confidence for children and adolescents, and
- Support youth and families experiencing negative impacts of the pandemic

Projet ECHO integrated **community-based participatory research with human centred design** to:

- actively engage and co-develop solutions with communities
- create and implement innovative, accessible and effective solutions that address community needs

Human-centered design phases for project ECHO

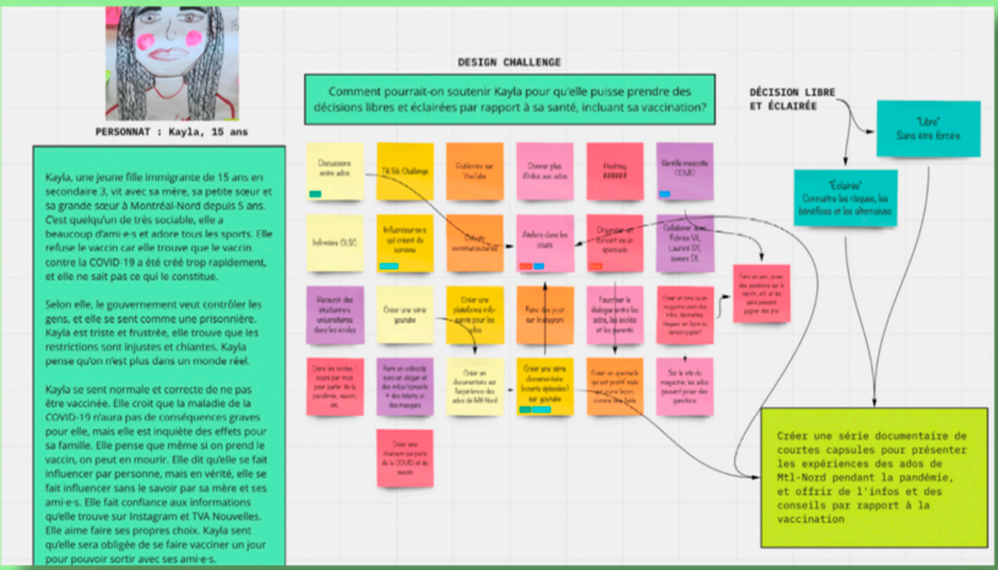

1 Understand

Community researchers conducted interviews with vaccine hesitant parents and youth to understand their needs and frame a design challenge (i.e., the problem they want to solve).

2 Ideate

Teams brainstormed ideas to create initial solutions to address their design challenges. They built prototypes (models of their solution), which were tested by gathering feedback from the community.

3 Implement

Teams created their final prototype and implemented it under real world conditions in the community. They collected data to understand if and how the solution created positive changes for users.

Chapter 2

02

Youths' Projects

2.1 Perspectives on COVID-19 vaccination
2.2 Les Ados du Nord: My Choice for My Community
2.3 Team APEX (ados of Parc Ex): Using educational gaming to combat misinformation

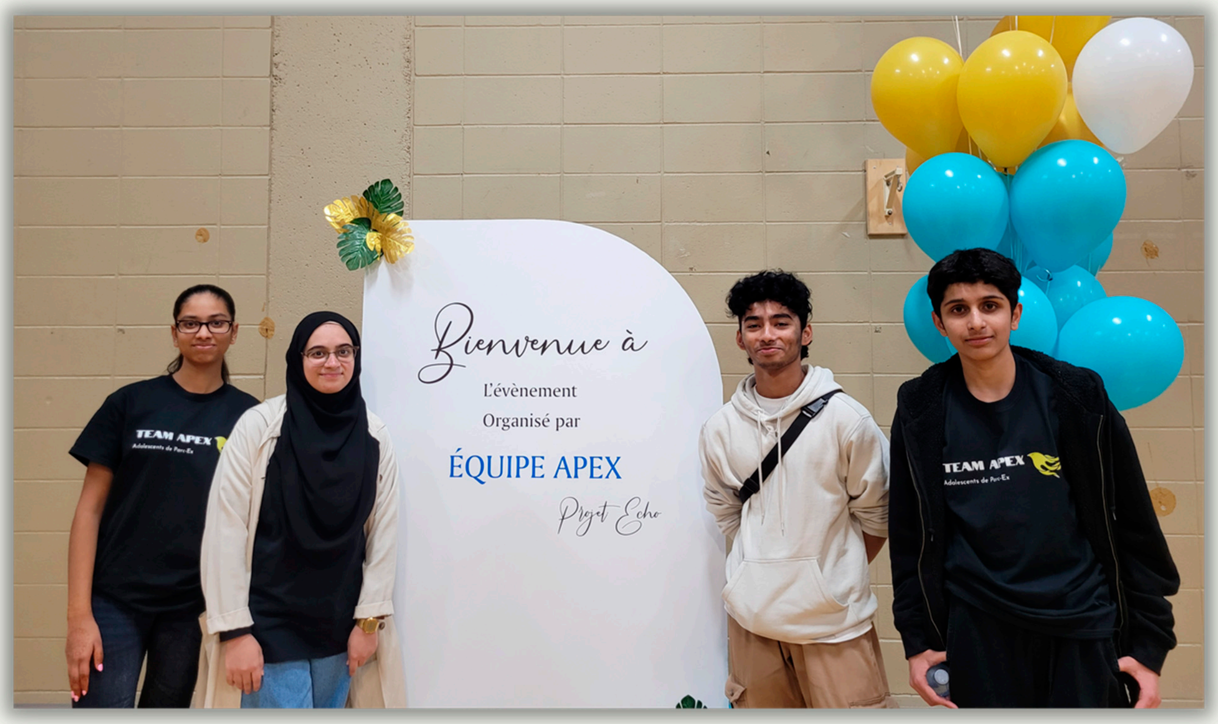

2.1 Youth Perspectives on Covid-19 Vaccination

The 8 youth community researchers conducted 25 interviews with non-vaccinated adolescents and 315 secondary students completed an online survey. Adolescents:

Have concerns about the safety, effectiveness, and necessity of COVID-19 vaccines

- "I've heard several stories about people having major side effects. People literally dying of the vaccine and pulmonary embolisms, diabetes getting worse."
- "I find it impossible to make a really good vaccine in like a year."
- "I am young and healthy, so the vaccine is not a priority for me"

Only 1 in 3 secondary students thinks COVID-19 vaccines are both safe and effective

Are not "anti-vax" and do not endorse conspiracy theories

- "There are some people that are really talking nonsense, like that the vaccine is controlled by the government, that there are chips in it. I don't know what, they're saying weird stuff, you gotta be careful."

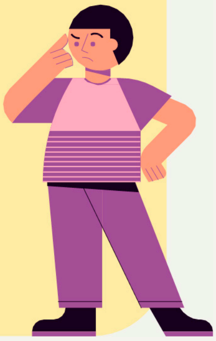

Do not want to feel pressured to get vaccinated

- "I think it's really unfair, because it's your body, your choice, and if you don't want to get the vaccine, that's your choice, and I don't understand why you should lose your everyday privileges just because you didn't get one dose or two. So I think it's really unfair."

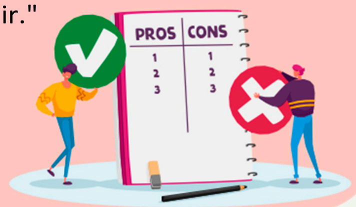

Want information they can trust to help them make decisions about vaccination

- "The measures taken against this pandemic are ridiculous. Each time they change the system, it really confuses me. I don't know who to believe anymore."
- "It's hard to find a vaccine for diseases and then like they were able to find for coronavirus fast like that"

Less than half of secondary students trust COVID-19 vaccine information from government sources (e.g., Health Canada, INSPQ) or scientific experts

Most adolescents in our study (both vaccinated and unvaccinated) had hesitations and questions about the novel COVID-19 vaccines but were unsure where to access trusted sources of information that would address their concerns. Team APEX and Les Ados du Nord used this information to design solutions to provide transparent, adolescent-friendly information. The following sections describe these projects!

2.2 <u>Les Ados du Nord</u>: My Choice for My Community

Les Ados du Nord conducted 11 semi-structured interviews with unvaccinated youth living in Montreal-Nord. Using their interviews, they created a persona named Kayla.

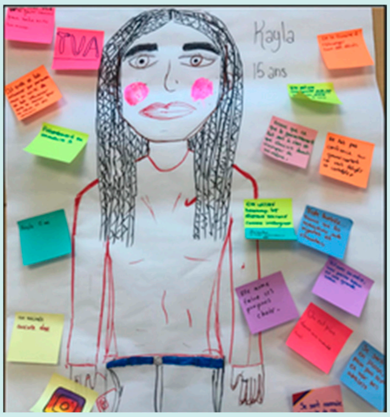

Kyla is 15 years old and she immigrated to Canada a couple years ago. With her family, she lives in Montreal North since 5 years. Kyla:

- believes the vaccine was developed too quickly and is worried about potential consequences of it
- believes the government wants to control people through the vaccine
- informs herself via Instagram and likes to making her own choices

Our design challenge

How might we support Kyla to make free and informed decisions about her health, including vaccination?

Our prototype

The prototype is a series of videos that :

- presents the experiences of Montreal Nord teens during the pandemic
- provides information about vaccination in a dynamic manner
- improves youth decision-making skills

This video series provides reliable information about COVID-19 vaccination to adolescents and promotes the sharing of youth experiences and .

Visit the link <u>here</u> to watch the series!

Notre série:
MON choix pour MA communauté

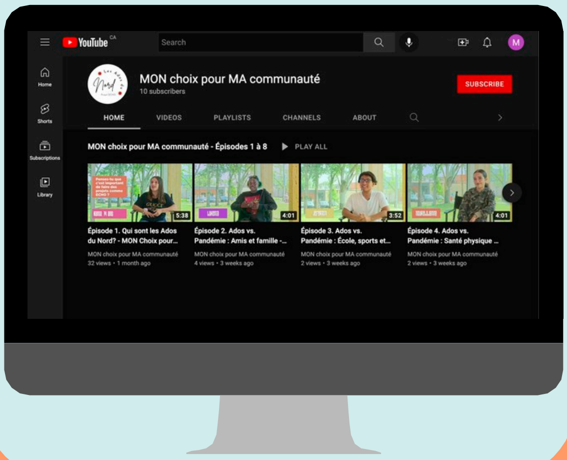

Content of the series: MON choix pour MA communauté

8 Episodes

1. Who are the Ados du Nord?
2. Ados vs. Pandemic : Friends & family
3. Ados vs. Pandemic : School, sports and field trips
4. Ados vs. Pandemic : Physical and mental health
5. Why are teens getting vaccinated?
6. Myth or reality? Discussion with Dr. Caroline Quach
7. Quiz info-vaccination : Discussion with Dr. Kate Zinszer
8. Making an informed decision

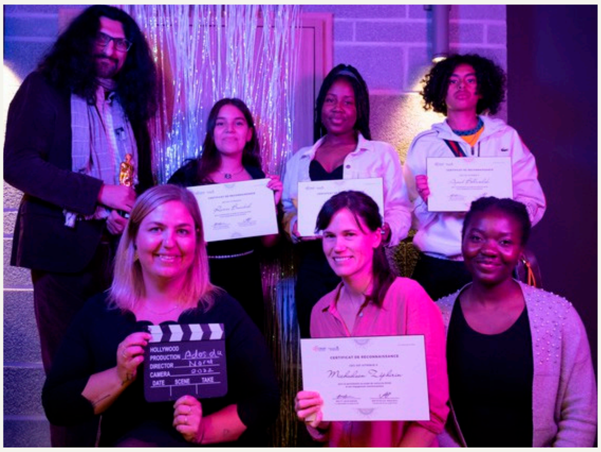

Impact of the series

Our solution

- Create a series of videos that focus on presenting the experiences and questions of Montreal-North teens
- Build the capacity of youth through creating a resource to help their decision-making

Anticipated Outcomes

- Improve the decision-making abilities of youth
- Encourage discussion among teens

Long-term impacts

- Increase vaccination rates among youth
- Reduce the impact of the pandemic in our community

The series was shared at a launch even in Montreal North and at a scientific congress (IIe congrès du CReSP).

2.3 Team APEX: Using educational gaming to combat misinformation

Team APEX conducted 13 semi-structured interviews with unvaccinated youth living in Parc-Extension. Using their interviews, they created a persona named Alex.

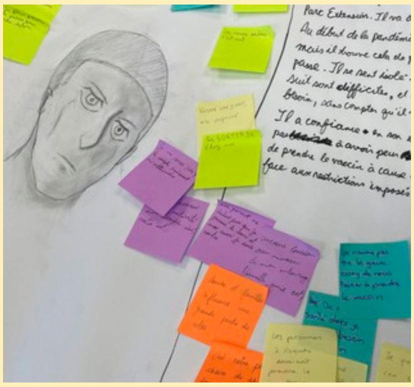

Our Persona: Alex

Alex is 16 years old and lives with his parents and 2 siblings. His parents got vaccinated against COVID-19 for their work, but advised him against getting vaccinated. He is:

- worried the vaccine may not be safe because it was developed too quickly
- confident in his health and believes the vaccine is unnecessary
- frustrated by the restrictions (vaccinal passport) placed on the unvaccinated by the government

Our design challenge

How might we support Alex to make better informed decisions during the next public health emergency?

Our prototype

Our prototype is an educational video game that :

- provides exercises to learn how to identify reliable sources of information
- is fun and relevant for the adolescent demographic
- empowers youth to collaborate in game development

The game SPECTHOV focuses on a specific aspect of decision-making: navigating available resources and making a judgement.

Scan the QR code to play the game!

The game: Projet SPECTHOV (Save the World, Conquer the Otherworldly Virus)

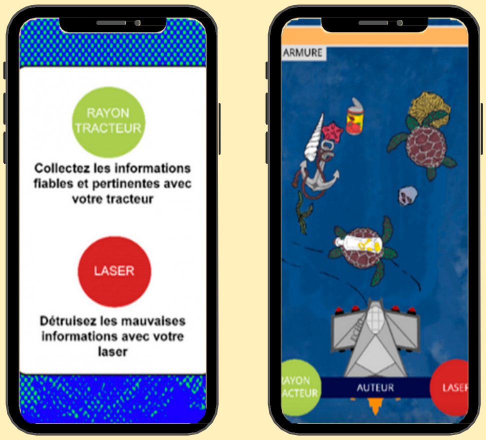

Our solution

- An educational game that focuses on teaching players different aspects of informed decision-making
- Build the capacity of youth through engagement in the game development process

Anticipated Outcomes

Improved health literacy including about COVID-19 vaccination and sources of misinformation

- Increased confidence of youth to make informed decisions during public health emergencies
- Contribute to youth empowerment

Long-term impacts

Lessons learned and next steps

We conducted 3 focus groups with youths in Parc-Extension to get their feedback on the game. Below are the lessons learned from conducting those focus groups.

1 Community pride

- The youth were proud that the game had been designed by their friends
- This translated into an enthusiasm to continue developing the game
- The youth liked the illustrations used in the game, other youth can also participate in future illustrations.

2 Language and accessibility

- Some words were not clear; the level of the French language used was difficult.
- At the moment, the game is not available in English or other languages
- The game does not work properly for everyone who tries to access it.

3 The solution to the needs identified by the youth?

- Youth suggested the game to be accompanied by other resources to answer their questions
- This game has the potential to be developed to better meet the needs of youth, for example, by having more levels, being more interactive and letting youth take the lead in its development. **We are currently looking for partners and funding to achieve these goals.**

Chapter 3

03

Parents' Projects

3.1 Perspectives on COVID-19 vaccination for their children
3.2 Vision of Parc Ex Mothers: Fostering community trust
3.3 ECHO du Nord parents: Bringing parents' voices into child health initiatives in Montreal-Nord

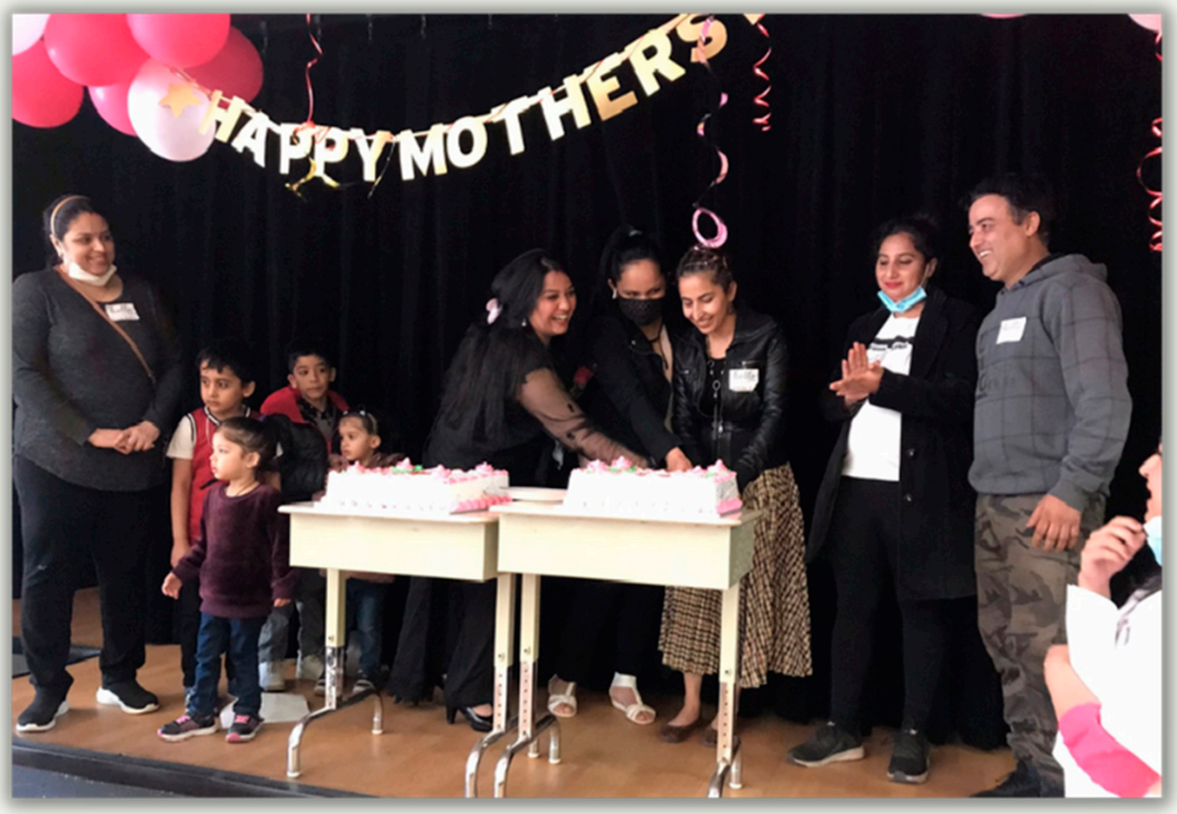

3.1 Parent Perspectives on Covid-19 Vaccination for Children

Parent researchers conducted 16 interviews with parents of unvaccinated children aged 5-11 and 145 parents of primary school children completed an online survey. Parents:

Lack confidence in the information they have received about COVID-19 vaccination for children

- "...[children] get sick like they would get sick from the flu or from anything else. [...] But also, from what they've always have been telling us. They have always been telling us kids are not being affected by COVID. And now I'm being asked to vaccinate them? I don't see why. I'm not convinced."

Have concerns about the safety, effectiveness, and necessity of COVID-19 vaccines for children

- "For me, it's the length of the time the vaccine has been studied. I don't want my kids to be the first ones, to be the population being tested on. It takes years to get results, to show long term impacts."

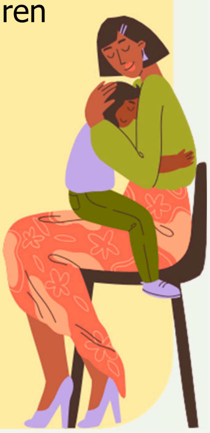

Got vaccinated for work, travel or vaccine passports, despite having concerns about the vaccine

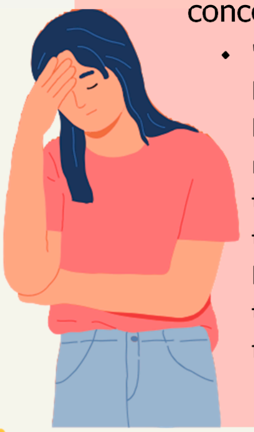

- "I got the vaccine, not because I agree with it, but because I have to. [...] The most important reason for me to be vaccinated is to have the right to travel once the borders reopen. If I don't have the vaccine, I can't go see my family."

Are overwhelmed and stressed by the impacts the pandemic has had on their lives and their families.

- "Well it was a big change from living a normal life being out every day socialising, going to work in the office, to being shut in, not going anywhere, not meeting your family nor your friends, being secluded from everything. Like, I don't know, it's as if you've been put into prison from one day to the other."

Parents reported receiving conflicting information about COVID-19 and vaccination for children, which made them lack confidence in public health recommendations. Many mothers also reported experiencing social isolation and emotional stress. They faced other hardships due to or not to the pandemic and consequently, vaccination was not a high priority for them. The two ECHO parent teams recognized the importance of supporting parents. The following sections describe their projects!

3.2 : The Vision of Parc-Ex Mothers: building community trust

The Vision of Parc-Ex Mothers conducted 11 semi-structured interviews with parents living in Parc-Extension and who had unvaccinated children between the ages of 5 and 11.

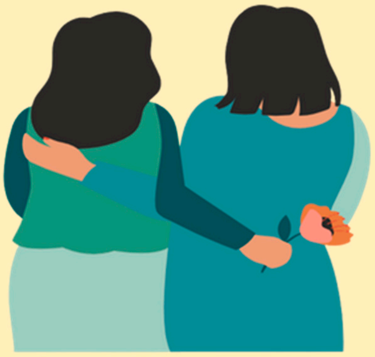

Our Persona: Zainab

Zainab, a 35 year-old, working mother of 2, is frustrated with the enduring restrictions of the pandemic, and wants a return to normalcy that does not put her children's health at risk.

She is confused and overwhelmed about the multiple changes in directives from the authorities and is tired of not knowing who to trust and which direction to take.

Our design challenge

How might we help Zainab access trustworthy sources of information so that she doesn't feel overwhelmed and anxious during her decision making process?

Our prototype

Our prototype is a community resilience group where:

- mothers can gather
- access resources and social networks that help and support them in the decision-making processes regarding health issues for their families (and themselves)

The team is committed to hold monthly meetings with approximately 20 mothers and discuss health or social topics of interest to them and organize other activities/events in the community. **We've held 5 meetups and 3 social events**.

Visualizing the group meetings

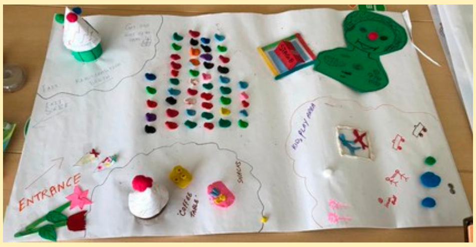

Meet-ups

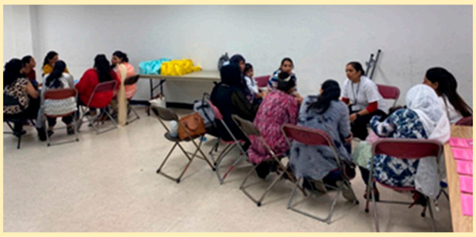

Our solution

- Create a resilience group for mothers in Parc-Ex that provides them with a safe space to discuss and have a support network
- Provide ressources, trainings, workshops that will empower mothers in many aspects of their lives and their children's.
- Multiple social determinants of health are taking into account for a holistic approach to health and well-being.

Anticipated Outcomes

A supportive of network of mothers in the community and an increased feeling of belonging

Long-term impacts

Improved agency among mothers and a feeling of empowerment to navigate the different systems in place (healthcare, immigration, etc.)

In design our solution, we were inspired by lessons and resources from the Mothers x Mothers project, a health meet up co-designed by DEO.org and Refugee Women's Network

Future Steps

1 Assess appreciation of the intervention by conducting focus groups and iterate based on participants recommendations

2 Ensure sustainability of the intervention beyond the ECHO project by:

- Joining a local community organization as a committee to pursue our actions as a community project run by mothers.
- Developing a curriculum based on topics of interest discussed during the pilot phase:
  - **Bloc 1: Physical health**
  - **Bloc 2: Mental health**
  - **Bloc 3: Professional development**
- Building partnerships and collaborations with community and institutional experts to facilitate the sessions. Searching for funding.

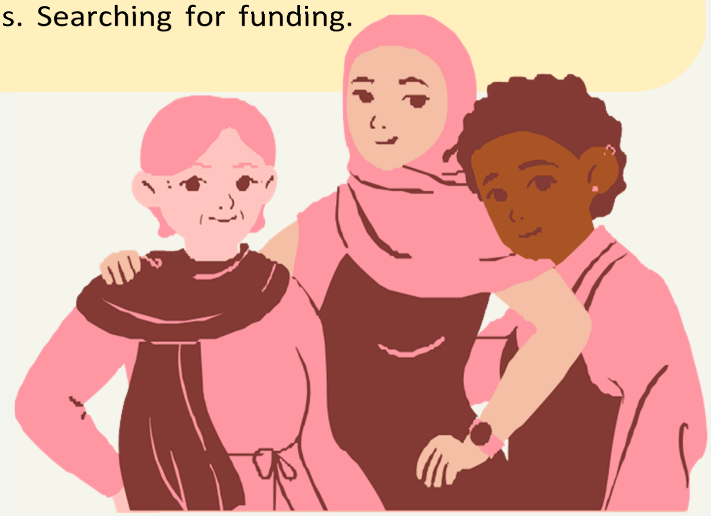

Strengths: Addressing Social determinants of health

Services are offered based on the needs of the mothers

- Meetings are held on Saturday to ensure mothers who work during the week can participate
- Supervised childcare
- Multilingual sessions

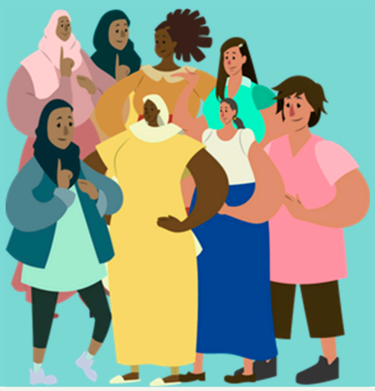

Creation of a supportive network

- Instructive sessions that allow them to break the isolation accentuated by the COVID-19 crisis
- Network with other mothers, and reduce their stress
- Access to clear and reliable information from experts

Limitations

COVID-19 Fatigue

- Considering the general fatigue of the population regarding the topic of COVID-19, vaccine hesitancy is, therefore, not the only subject addressed
- The solution's impact on COVID-19 vaccination uptake cannot be measured in the very short term.

It is crucial to recognize and address the urgent needs of communities facing multiple hardships, and to maintain the dialog between them and experts to be able to communicate effectively with them in the event of future crises.

Missing participants

- Only mothers who have not vaccinated their children were interviewed, thereby excluding mothers who have done it while being hesitant, which could translate into non-adherence to taking future doses
- However, the mothers' group sessions are open to all, regardless of the children vaccination status. Information are accessible to all, and we continue learning from the participants and adapting the intervention to them.

Isolated mothers and men in the community

- We recognize that women who asylum seekers or newcomers to Canada may be temporarily distant from our community groups due to the experience of immigrating to a new country, therefore efforts need to be made to reach them directly.
- There is a need for solutions that involve fathers as they are key contributors and decision makers in some households.

3.3 : ECHO du Nord parents: Bringing parents' voices into child health initiatives in Montreal-Nord

The ECHO du Nord parents conducted 6 semi-structured interviews with parents living in Montreal-Nord and who had unvaccinated children between the ages of 5 and 11.

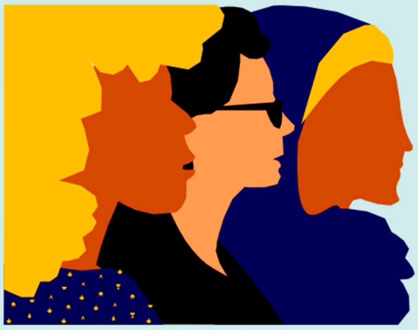

Our Persona: Sara

Zainab, a 40 year-old, mother of 3, is stressed about the loss of income due to the pandemic. She is worried that she will not be able to meet her family's needs. She is:

- anxious as she doesn't know who to turn to to get reliable information on the vaccine
- afraid of making the wrong decision, which could jeopardize her children's health in the future
- afraid of being a bad mother who can't protect what is most precious to her

Our design challenge

How might we help Sara feel like a respected and protected member of her community; and access reliable sources of information to make decisions about her children's health?

Our prototype

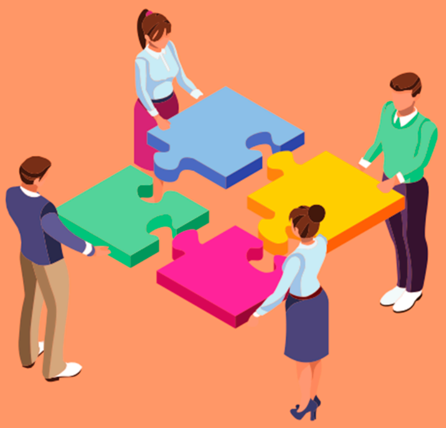

Our prototype aims to

- offer parents the opportunity to have their voices heard in the context of a community collective action project on health issues of concern to families in Montreal North
- work in partnership with existing organizations in Montreal Nord

After reassessing our capacities as a group, we opted to **defer our project to an organization that already works on the ground in Montreal-North.** For this reason, and to stay faithful to our persona, we decided to **start a call for proposals, and select the best candidate that will be able to reach our goals.**

Our solution

Provide a $15,000 grant to support a Montreal Nord organization that aligns with ECHO du Nord to involve the voices of parents in matters relating to their children's health

Anticipated Outcomes

- Parent participation in existing projects
- Increased access to reliable information

Long-term impacts

- Mitigation of social determinants of health in Montreal Nord
- Enhanced quality of life

What we learned about our community

The feeling of being a **"lab rat" is often debilitating and a main reason behind the non-adherence such as vaccine hesitation during a global pandemic.** Our research showed that having their voices heard by local decision-makers, knowing that their fears are really taken into consideration and having clear answers to their real questions are key factors to reinforce parents' trust.

We have tested with seven parents, the prototype of a Parents' Health Council in Montreal-North, in charge of organizing sessions to collect and relay to local decision-makers, parents' questions, concerns and recommendations concerning various health issues affecting their children and their families. **The issues of interest to parents were the lack of safety, alcohol and drug use, nutrition, smoking, physical activity, sexuality and vaccination of children against COVID-19.** Because our team did not have the skills and resources, we decided to launch a call for proposals to organizations in Montreal-North that align to this vision. We issued the call for proposals in July 2022. Unfortunately, no applications from community organizations were submitted. It is possible to read the presentation document and application form on our project webpage: ECHO du Nord — ECHO (projet-echo-mtl.ca)

Future Steps

1. Prepare a narrated PowerPoint on our process and interviews results and share it with participants and Montréal-Nord community organizations

2. Conduct phone interviews with Montréal-North community organizations to understand why applications were not submitted.

Chapter 4

04

Reflections & Recommendations

4.1 Project strengths
4.2 Challenges
4.3 Conclusions

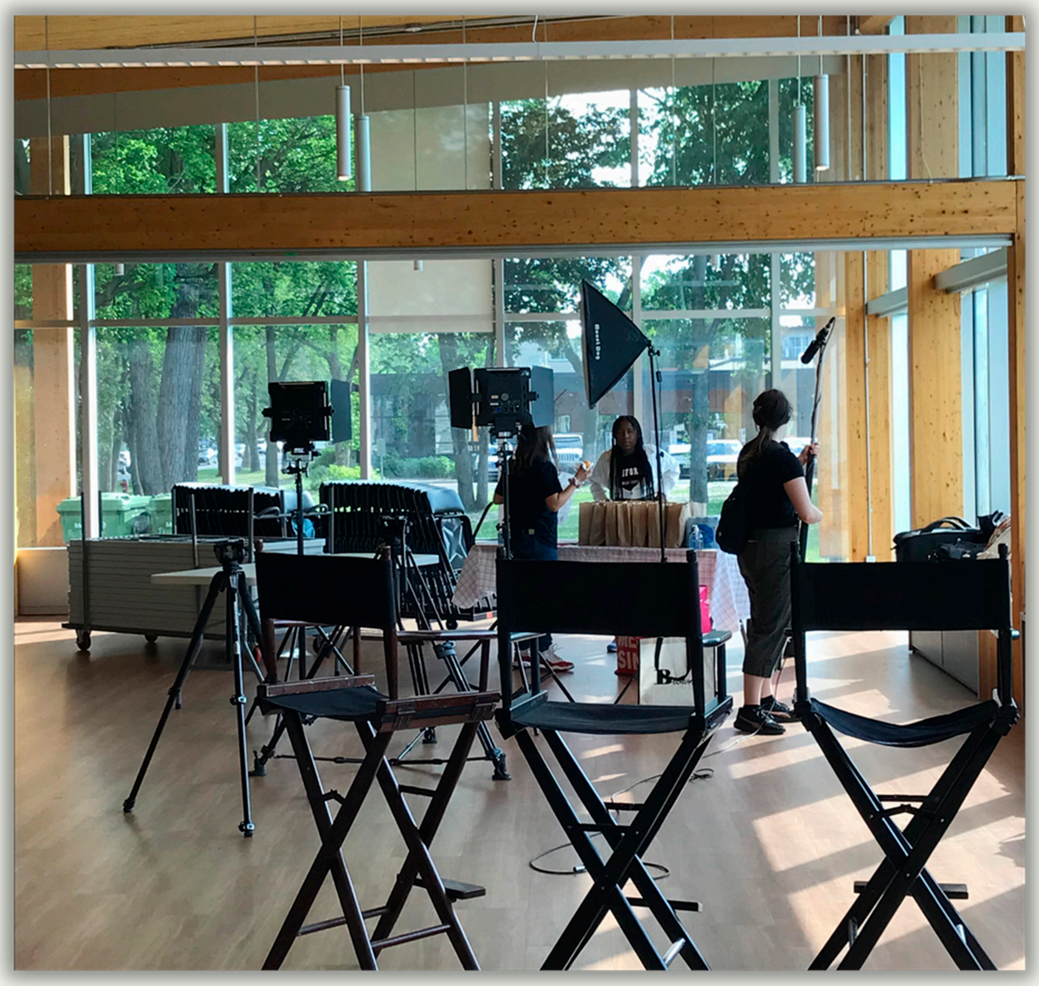

4.1 : Project strengths

• A diverse team with opportunities for learning, creativity, and collaboration

"Yes, well, I'm going to go more on a personal level, in terms of how the project went. What I really liked and what I told my colleagues at the beginning was the diversity in our team. Whether it's in terms of our professional backgrounds, or in terms of our approaches. [...] So that's interesting. Culturally we are also different. We also have gender parity. I loved that. In my life, in general, I don't always have this diversity around me, so I really liked that." Community researcher – Parent

• A diverse team with opportunities for learning, creativity, and collaboration

"And the second thing, I became popular. Wherever I walk, everybody knows me. So everybody knows me everywhere. Hello, hello, hello. So it gave me pride and most of them know that I'm doing something good, even when they share their opinion with me, they say you have to tell them we need this, we need that and thank you, thank you. I really enjoyed it and I learned a lot from my community as well." Community researcher - Parent

"Well, the thing I liked the most was interviewing teenagers. When we did little interviews on Zoom, it helped me a lot to be less shy, to be open-minded, to understand people of my age [...]" Community researcher - Adolescent

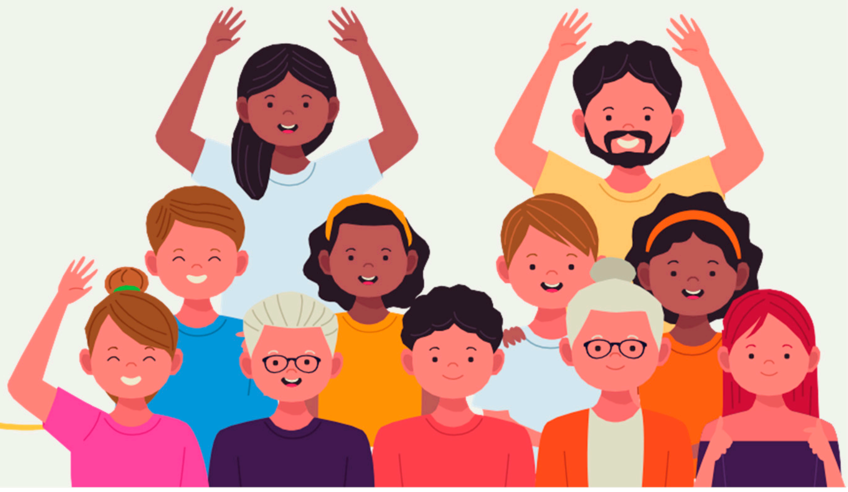

4.2 : Challenges

Despite the implementation of three promising projects, we have encountered several challenges, the most important of which are:

- ## Working remotely

Human centred design is ideally a hands-on process, with an emphasis on in-person interviews, group brainstorming and prototyping using art materials. Due to the COVID-19 pandemic, however, we often had to work remotely. Community researchers expressed difficulties in connecting with participants and local community organizations during the needs assessment phase of the study.

- ## Recruitment challenges specific to COVID-19 vaccination

Several people who were not vaccinated or who had not vaccinated their children against COVID-19 expressed concern about participating due to stigma around non-vaccination. Despite our guarantee of confidentiality, some refused to participate in the interviews for this reason, while a few were remaining reserved or vague in their responses during interviews.

- ## The rapidly changing context of the pandemic

The project had to adjust to the emergence of the Omicron variant and the lifting of the vaccine passport policy, both factors that affected vaccine decision-making. With this changing landscape, the project's HCD approach was flexible enough that the community projects were able to expand from a narrow focus on vaccination to a broader focus on health literacy and decision making.

- ## Difficulties conducting research-action in Montreal-North vs Parc-Extension

1. Due to the presence of University of Montreal's School of Public Health in Parc-Extension,

·many community and citizen organizations have experience collaborating with research projects. The following reasons facilitate needs assessment and pilot projects' implementation in the neighbourhood: (1) the excellent knowledge of the actors in the field, (2) the presence of resource persons capable of linking the research team with potential collaborators, participants and volunteers, and (3) the direct involvement of some community researchers in local organizations. The downside, however, is that citizens were over-solicited by research projects, including many on COVID-19, which resulted in difficulties in recruiting interview participants.

2. Several organizations told us that the reputation of the Université de Montréal and its projects were eroding in Parc-Extension because of gentrification issues, which many hold the expansion of the institution responsible, and non-winning collaboration practices for community organizations in several research projects.

3. As for Montréal-Nord, the borough is almost 7 times larger than Parc-Extension (11.1 km^2 vs 1.6 km^2). In addition, academic and community researchers were less familiar with the field actors, which made it more difficult to collaborate, implement and assess solutions, and share results.

- ## Ensuring interventions sustainability beyond the research project

The ECHO project will end in December 2022. We are currently working to ensure sustainable structures for the 3 pilot projects and defining detailed plans for the next year and obtaining funding so that they can continue under the responsibility of community actors. As such, Vision of Parc-Extension Mothers and Team APEX are both looking to become local community organization committees to continue the development and implementation of their projects. Les Ados du Nord would like to transmit their project to schools in Montreal so that they can conduct discussion sessions with the videos and guidebook produced.

Conclusions

1. Parents and adolescents, through their community researcher's role, acquired research skills, design, and pilot a solution for their communities.

2. The "by the community for the community" approach was an empowering experience for all, especially for the adolescents.

3. Human Centred Design is a creative process that is hard to adapt to remote working.

4. In Parc-Extension we recognize that actions must be taken to ensure good relations between researchers and community stakeholders. Conducting a study on local community organizations' perceptions of Université de Montréal and its research projects would allow for clear courses of action on the subject.

We thank you for your ongoing support of the ECHO project

Acknowledgements

- Parents and youth of Montreal North and Parc-Extension who participated in our surveys and interviews
- Participating schools: Lucien Pagé, Barthélémy-Vimont, Sinclair-Laird, Camille-Laurin, Henri-Bourassa, René-Guénette, Sainte-Colette, Saint-Vincent-Marie and Gérald McShane, and their school boards (CSSDM, CSSPI, EMSB)
- Community organizations in Montreal-Nord and Parc Extension: ACCESSS, Afrique au féminin, Centre d'Action Bénévole de Montréal-Nord, Éco-quartier Montréal-Nord, Jeunesse Unie, Les fourchettes de l'espoir,
- Community-based Action Research (CBAR) Network in Parc-Extension, CoVivre program
- Community and subject-matter experts: Sasha Dyck, Ashley Vandermorris, Cécile Rousseau, Ève Dubé, Laurence Monnais, Roxane de la Sablonnière, Ananya Banerjee, Caroline Quach, who accompanied the teams in contextual analysis of their results

MANY THANKS TO ALL

We look forward to continued collaboration!

QUESTIONS? COMMENTS? IDEAS?

Learn more about the project and read our blog at:

www.projet-echo-mtl.ca

Contact us:

Email:
Krystelle.marie.abalovi@gmail.com

CIHR IRSC Canadian Institutes of Health Research Instituts de recherche en santé du Canada

CReSP Centre de recherche en santé publique

Humanos DESIGN THINKING FOR HUMANITY
